# Editorial: engineering approaches to study cardiovascular physiology: modeling, estimation, and signal processing

**DOI:** 10.3389/fphys.2012.00425

**Published:** 2012-11-05

**Authors:** Zhe Chen, Riccardo Barbieri

**Affiliations:** ^1^Neuroscience Statistics Research Lab, Department of Anesthesia Critical Care and Pain Medicine, Massachusetts General Hospital, Harvard Medical SchoolBoston, MA, USA; ^2^Department of Brain and Cognitive Sciences, Massachusetts Institute of TechnologyCambridge, MA, USA

With cardiovascular diseases being among the main causes of death in the world, quantitative modeling, assessment and monitoring of cardiovascular dynamics, and functioning play a critical role in bringing important breakthroughs to cardiovascular care. Quantification of cardiovascular physiology and its control mechanisms from physiological recordings, by use of mathematical models and algorithms, has been proved to be of important value in understanding the causes of cardiovascular diseases and assisting the diagnostic and prognostic process. This E-Book is derived from the *Frontiers in Computational Physiology and Medicine* Research Topic entitled “Engineering Approaches to Study Cardiovascular Physiology: Modeling, Estimation and Signal Processing.” Its goal is to bring established experts together in order to present a sample of state-of-the-art studies in cardiovascular physiology, and to give a general idea of the very different approaches that can be adopted to answer the research challenges posed by the varied, complex nature of the cardiovascular system. This book presents 10 contributions, in the form of review and original research articles.

There are two review articles. The first review article by Chen et al. (2012) presents a unified point process probabilistic framework to assess heart beat dynamics and autonomic cardiovascular control. Using clinical recordings of healthy subjects during Propofol anesthesia, the authors demonstrate the effectiveness of their approach by applying the proposed paradigm to estimate instantaneous heart rate (HR), heart rate variability (HRV), respiratory sinus arrhythmia (RSA) and baroreflex sensitivity (BRS). The second review article, contributed by Zhang et al. (2011), provides a comprehensive overview of tube-load model parameter estimation for monitoring arterial hemodynamics. The authors discuss the motivation, assumption and validity of the proposed tube-load model, and summarize various estimation techniques and their experimental results, as well as potential applications.

The remaining eight original research articles can be mainly classified into two categories. The two articles from the first category emphasize modeling and estimation methods. In particular, the paper “Modeling the autonomic and metabolic effects of obstructive sleep apnea: a simulation study” by Cheng and Khoo (2012), combines computational modeling and simulations to study the autonomic and metabolic effects of obstructive sleep apnea (OSA). The second paper, “Estimation of cardiac output and peripheral resistance using square-wave-approximated aortic flow signal” by Fazeli and Hahn (2012), presents a model-based approach to estimate cardiac output (CO) and total peripheral resistance (TPR), and validates the proposed approach via *in vivo* experimental data from animal subjects.

The six articles in the second category focus on application of signal processing techniques and statistical tools to analyze cardiovascular or physiological signals in practical applications. the paper “Modulation of the sympatho-vagal balance during sleep: frequency domain study of heart rate variability and respiration” by Cabiddu et al. (2012), uses spectral and cross-spectral analysis of heartbeat and respiration signals to assess autonomic cardiac regulation and cardiopulmonary coupling variations during different sleep stages in healthy subjects. the paper “increased non-gaussianity of heart rate variability predicts cardiac mortality after an acute myocardial infarction” by Hayano et al. (2011) uses a new non-gaussian index to assess the HRV of cardiac mortality using 670 post-acute myocardial infarction (AMI) patients. the paper “non-gaussianity of low frequency heart rate variability and sympathetic activation: lack of increases in multiple system atrophy and parkinson disease” by Kiyono et al. (2012), applies a non-gaussian index to assess HRV in patients with multiple system atrophy (MSA) and parkinson diseases and reports the relation between the non-gaussian intermittency of the heartbeat and increased sympathetic activity. The paper “Information domain approach to the investigation of cardio-vascular, cardio-pulmonary, and vasculo-pulmonary causal couplings” by Faes et al. (2011), proposes an information domain approach to evaluate nonlinear causality among heartbeat, arterial pressure, and respiration measures during tilt testing and paced breathing protocols. The paper “integrated central-autonomic multifractal complexity in the heart rate variability of healthy humans” by Lin and Sharif (2012), uses a relative multifractal complexity measure to assess HRV in healthy humans and discusses the related implications in central autonomic interactions. Lastly, the paper “Time scales of autonomic information flow in near-term fetal sheep” by Frasch et al. (2012), analyzes the autonomic information flow (AIF) with kullback–leibler entropy in fetal sheep as a function of vagal and sympathetic modulation of fetal HRV during atropine and propranolol blockade.

In summary, this Research Topic attempts to give a general panorama of the possible state-of-the-art modeling methodologies, practical tools in signal processing and estimation, as well as several important clinical applications, which can altogether help deepen our understanding about heart physiology and pathology and further lead to new scientific findings. We hope that the readership of *Frontiers* will appreciate this collected volume and enjoy reading the presented contributions. Finally, we are grateful to all contributed authors, reviewers, and editorial staffs who had all put tremendous effort to make this E-Book a reality.
